# Cervical cancer incidence, mortality, and burden in China: a time-trend analysis and comparison with England and India based on the global burden of disease study 2019

**DOI:** 10.3389/fpubh.2024.1358433

**Published:** 2024-03-06

**Authors:** Siyu Wu, Jun Jiao, Xiaoyu Yue, Yaping Wang

**Affiliations:** Department of Gynecology and Obstetrics, Qilu Hospital (Qingdao), Cheeloo College of Medicine, Shandong University, Qingdao, China

**Keywords:** cervical cancer, incidence, mortality, disease burden, projection, China, England, India

## Abstract

**Background:**

Cervical cancer is the fourth highest incidence of malignancy in the world and a common cause of cancer death in women. We assessed the trends of incidence and mortality and disability-adjusted life year (DALY) in China, England and India from 1990 to 2030.

**Method:**

Data were obtained from the Global Burden of Disease (GBD) database. We collected the number and rate of incidence, death and DALY from 1990 to 2019 and calculated the estimated annual percentage change (EAPC). Further analysis was carried out by ages and years. We also collected attributable risk factors to cervical cancer. Finally, we utilized the Bayesian Age-Period-Cohort (BAPC) model to forecast trends in the rate of age-standardized incidence (ASIR) and age-standardized death (ASDR) the for the next decade.

**Result:**

Globally, the incidence of cervical cancer cases increased from 335,641.56 in 1990 to 565,540.89 in 2019. In 2019, the ASIR and ASDR of cervical cancer were higher than those of India but lower than those of England. Furthermore, unsafe sex and smoking emerge as prominent risk factors for cervical cancer. Over the next decade, ASIR and ASDR are expected to decline in China and England, while India’s ASIR is still on an upward trend and ASDR is on a downward trend.

**Conclusion:**

The epidemiological data of cervical cancer in these three countries reflects the influence of different stages of development and healthcare systems. Trends over the next decade suggest that China and India still face a huge burden of cervical cancer. When England has made significant progress, China and India need to take more measures to improve the prevention and control of cervical cancer.

## 1 Introduction

Cervical cancer is the fourth most common malignancy in the world and a common cause of cancer death in women, with approximately 604,000 new cases and 342,000 deaths worldwide in 2020 (1). Cervical cancer has the highest incidence among young women and is typically categorized into two primary histological types: adenocarcinoma and squamous cell carcinoma (2). During its early stages, cervical cancer often remains asymptomatic. However, as the disease progresses, it may manifest with noticeable symptoms, including abnormal vaginal bleeding, pelvic discomfort, pain during sexual intercourse, and an unusual vaginal discharge. Cervical cancer is caused primarily by persistent infection with high-risk types of human papillomavirus (HPV) (3), which is a common sexually transmitted infection. Most women are exposed to HPV at some point in their life, but in many cases, the immune system clears the infection. However, in some instances, the virus can persist and lead to changes in the cervical cells, potentially progressing to cervical cancer over time. Unfortunately, while the HPV vaccine and cervical cancer screening have become more widely available, the incidence of cervical cancer has not decreased significantly.

Cervical cancer is a growing global burden for both developing and developed nations. Furthermore, in 2020, the World Health Organization (WHO) initiated a far-reaching appeal to all nations across the globe, urging them to access resources with the aim of expediting the eradication of cervical cancer as a critical public health issue (4). In the United States, approximately 100,000 individuals continue to receive treatment for precancerous cervical lesions. Out of those, 14,000 are newly diagnosed with cervical cancer, and tragically, 4,000 have lost their lives to this disease (5). An observational study revealed that, in relation to both incidence rates and mortality, regions with a high socio-demographic index (SDI) exhibited significantly lower figures compared to regions with a low SDI (6). While the Chinese government has initiated free HPV vaccination programs for adolescents and provided free cervical cancer screenings for women aged 35 to 64 years in rural areas, the incidence of cervical cancer in China remains on the rise (7). Due to its substantial population, China accounted for 11.9% of global cervical cancer deaths in 2017, contributing to 12.3% of the worldwide DALY due to cervical cancer (8).

The Global Burden of Disease (GBD) 2019 conducted an evaluation of 369 causes of mortality or injury and 87 risk factors across 204 countries and regions. Through the GBD database, we can access a wealth of data, including prevalence and incidence of diseases, mortality, disability-adjusted life years (DALYs), years of life lost (YLL), and years lived with disability (YLD). In this study, we utilized the GBD 2019 database to analyze and compare trends in cervical cancer-related burdens across three countries: China, India, and England, spanning from 1990 to 2019. Additionally, we projected the age-standardized incidence rate (ASIR) and age-standardized death rate (ASDR) for cervical cancer for the years 2020 to 2030. The findings from this analysis will aid in predicting longitudinal changes in cervical cancer incidence. We conducted a comparative analysis of data from China, England (a developed country), and India (a developing country) to examine the epidemiology data of cervical cancer. It investigated the influence of different stages of development and healthcare systems, offering a valuable reference and guidance for formulating policies to reduce the burden of this disease in the near future.

## 2 Method

### 2.1 Data sources

The methodology of the GBD Study 2019 has been described elsewhere (9). We have extracted cervical cancer data and demographic information from China, England and India from 1990 to 2019 from an online database.^1^ We have gathered data for incidence, death, and DALY numbers over a 30-year period, along with data for age-specific and age-standardized incidence, death, and DALY rates. Furthermore, we have collected information on risk factors attributable to cervical cancer. Ethics approval and informed consent were not required for this study because of public accessibility of the data. This study followed the Guidelines for Accurate and Transparent Health Estimates Reporting (GATHER) for cross-sectional studies (10).

### 2.2 Statistical analysis

We collected the number and age-standardized rate of incidence and death, along with the 95% uncertainty interval (UI) obtained from the database. Temporal trends in the ASIR and ASDR from 1990 to 2019 were quantified by the estimated annual percentage change (EAPC) to assess the burden of cervical cancer. The EAPC serves as a commonly utilized metric for assessing ASR trends within defined time intervals (11). If both the EAPC and its 95% CI lower limit were > 0, it is considered that the ASR is exhibiting an increasing trend. Conversely, if both <0, it is deemed that the ASR is in a declining trend. Otherwise, the ASR is regarded as stable. We also analysed the number and age-standardized rate of incidence, mortality and burden of cervical cancer in 2019 by different age groups to find the age-specific patterns. Then, we calculated and reported the incidence, death and DALYs attributable to all risk factors, including unsafe sex and smoking in China, England and India in the last 30 years. Finally, we used the Bayesian age-period-cohort analysis (BAPC) model with integrated nested Laplace approximation (INLA) to predict the trends in the ASIR and ASDR from 2020 to 2030 in three countries. Then we used the GBD Foresight Visualization to verify the results.

The data analysis was conducted using the R program (version 4.3.1) and RStudio. The BAPC prediction model utilized the software packages “nordpred (version 1.1),” “BAPC (version 0.0.36),” and “INLA (version 23.09.09).” Graphs were generated using GraphPad Prism (version 9.0.0) and the R.

## 3 Results

### 3.1 Incidence and mortality of cervical cancer

Globally, the incidence of cervical cancer cases increased by 68.50% from 335,641.56 in 1990 to 565,540.89 in 2019. During the same period, the ASIR decreased by an average of 0.38% (95% CI 0.34–0.41%) per year, dropping from 14.91 per 100,000 in 1990 to 13.35 per 100,000 in 2019 (Supplementary Figure 1A). The ASDR also decreased by an average of 0.93% (95% CI 0.88%−0.98%) per year from 8.48 in 1990 to 6.51 in 2019 (Supplementary Figure 1B). Although the ASIR has declined, the overall global burden of disease remains high.

In China, there was a significant increase in the incidence of cervical cancer cases, which increased by 169.80% from 40,680.98 in 1990 to 109,759.90 in 2019. The ASIR in China increased by an average of 1.61% (95% CI 1.36–1.86%) per year during the same period, going from 8.41 per 100,000 in 1990 to 11.01 per 100,000 in 2019.

Conversely, England saw a decrease in the incidence of cervical cancer cases, which declined from 4,236.1 in 1990 to 2,827.45 in 2019, with an EAPC of-1.89%. In India, there was an increase in the number of incidence cases, rising from 47,408.5 in 1990 to 84,981.9 in 2019. However, the ASIR in India decreased from 16.65 per 100,000 in 1990 to 13.1 per 100,000 in 2019 (Table 1).

**Table 1:** The Incidence numbers, age-standardized incidence, and temporal trends of cervical cancer from 1990 to 2019.

Characteristic	1990		2019		1990–2019
	Numbers No. (95% UI)	ASR per 100,000 No. (95% UI)	Numbers No. (95% UI)	ASR per 100,000 No. (95% UI)	EAPC (95% UI)
Global	335641.56 (300354.25–393893.3)	14.91 (13.37–17.55)	565540.89 (481523.96–636434.7)	13.35 (11.37–15.03)	−0.38 (−0.41–0.34)
China	40680.98 (30919.5–73182.36)	8.41 (6.44–15)	109759.9 (58188.68–141538.97)	11.01 (5.87–14.22)	1.61 (1.36–1.86)
India	47408.5 (37133.95–60574.48)	16.65 (13.2–21.45)	84981.9 (65941.69–110275.84)	13.1 (10.18–17.09)	−1.07 (−1.29–0.85)
England	4236.1 (3521.88–4389.26)	13.67 (10.65–14.22)	2827.45 (2145.29–3721.08)	7.57 (5.71–10)	−1.89 (−2.17–1.61)

As shown in Table 2, the ASDR has decreased in China, India, England, and globally. Nevertheless, the number of deaths continued to climb in both China and India, while it declined in England. Specifically, the ASDR decreased from 5.85 in 1990 to 5.13 in 2019, with an EAPC of 0.09%. In India and England, the ASDR decreased from 10.9 and 5.64 in 1990 to 7.38 and 2.74 in 2019, respectively.

**Table 2:** The death numbers, age-standardized death rate, and temporal trends of cervical cancer from 1990 to 2019.

Characteristic	1990		2019		1990–2019
	Numbers No. (95% UI)	ASR per 100,000 No. (95% UI)	Numbers No. (95% UI)	ASR per 100,000 No. (95% UI)	EAPC (95% UI)
Global	184527.08 (164835.85–218942.1)	8.48 (7.59–10.07)	280479.04 (238863.96–313929.7)	6.51 (5.55–7.29)	−0.93 (−0.98–0.88)
China	26419.82 (20524.28–43527.4)	5.85 (4.59–9.57)	53441.4 (30398.79–68856.44)	5.13 (2.92–6.6)	0.09 (−0.17–0.34)
India	27896.8 (22139.81–35328.05)	10.9 (8.59–13.74)	45446.63 (35004.18–62351.78)	7.38 (5.71–10.13)	−1.6 (−1.81–1.4)
England	2110.13 (1958.81–2186.3)	5.64 (5.13–5.81)	1342.41 (1221.76–1870.47)	2.74 (2.54–3.72)	−2.39 (−2.67–2.11)

EAPC, estimated annual percentage change.

### 3.2 Temporal trends of age-standardized incidence, mortality and DALY rate of cervical cancer

**Figure 1:**
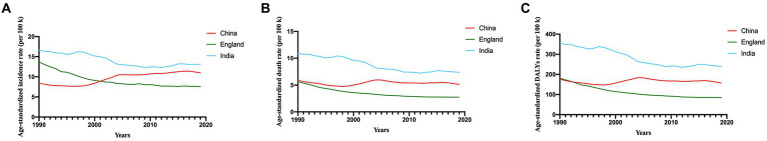
Time trends of age-standardized incidence **(A)**, mortality **(B)**, and DALY **(C)** rates of cervical cancer in China, England and India. DALY, disability-adjusted life-years. k, thousand.

Since 1990, the ASIR has gradually increased until 2019 in China, in contrast to England, where the ASIR has exhibited a continuous decline over the same period (Figure 1A). India’s ASIR consistently surpasses that of both China and England, although it also demonstrates a slow decreasing trend. Notably, India had the highest ASDR among these three countries, while England had the lowest (Figure 1B). Both India and England show declining trends in ASDR, whereas China’s pattern appears to fluctuate. The trends for DALY rates and ASDR follow a similar pattern (Figure 1C). The burden of disease in China is slowly increasing, in India it is consistently high, although it is declining, and in the England, it has remained low and falling.

### 3.3 The incidence, mortality and burden of cervical cancer in 2019 by age

**Figure 2:**
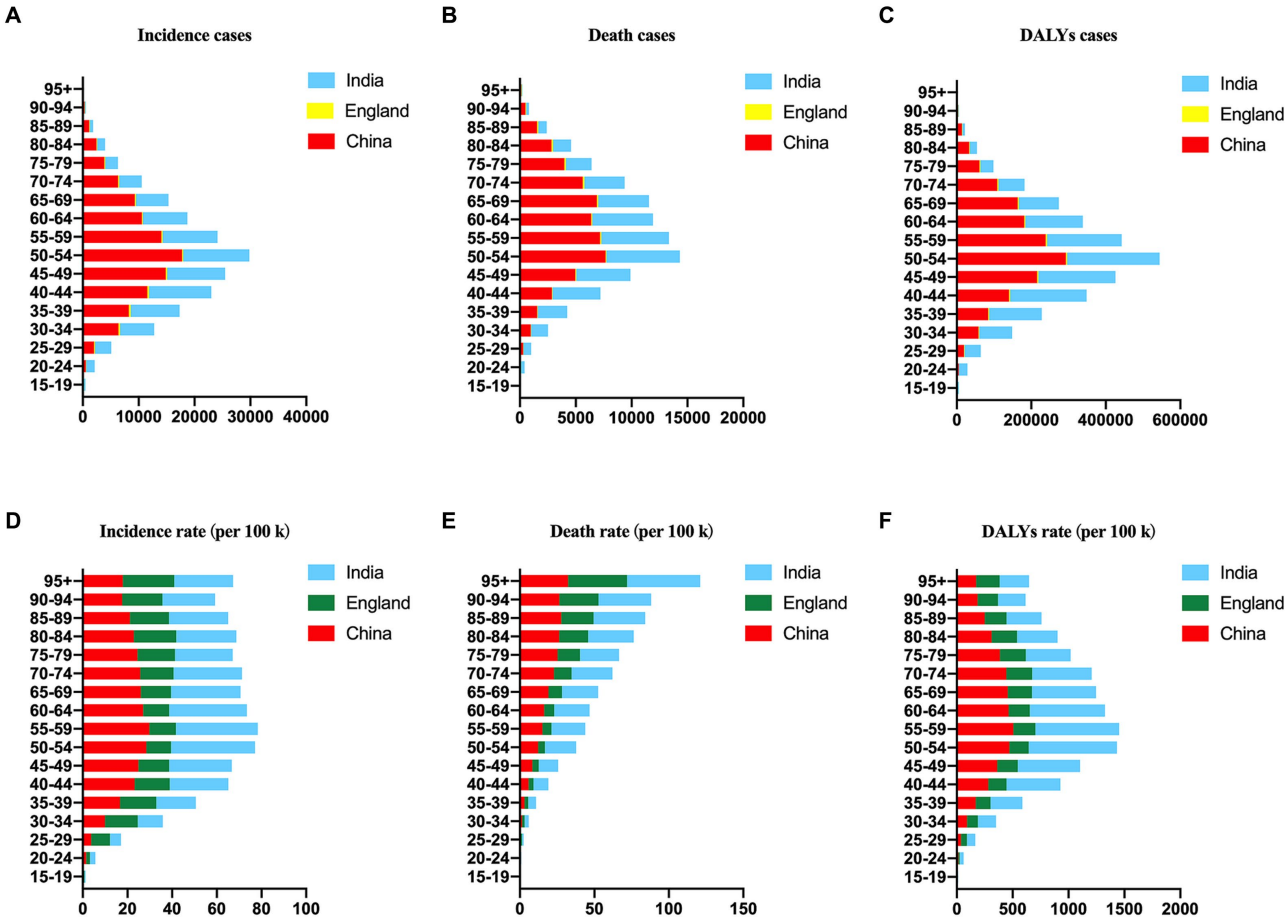
The number and rate of age-standardized incidences **(A,D)**, deaths **(B,E)**, and DALYs **(C,F)** of cervical cancer in China, England and India in 2019 by age.

The incidence, mortality and burden of cervical cancer in China, England and India in 2019 by different age groups were shown in Figure 2. Notably, the incidence of new cases reached its peak in the 50–54 age group in China and India, whereas in England, it was highest in the 35–39 age group (Figure 2A). Similarly, the number of deaths peaked in the 50–54 age group in China and India, while in England, it was most prominent in the 70–74 age group (Figure 2B). The DALY numbers in India and China were greater than that in England (Figure 2C).

Furthermore, the ASIR reached its pinnacle at 29.83 in the 55–59 age group in China, 37.64 in the 50–54 age group in India, and 18.94 in the 80–84 age group (Figure 2D). Conversely, the ASDR displayed an increasing trend with age and reached its maximum in the 95+ age group in China, India, and England, with values of 32.43, 49.04, and 39.52, respectively (Figure 2E). The DALY rates peaked at 506.33 in the 55–59 age group in China, 789.56 in the 50–54 age group in India, and 231.12 in the 70–74 age group in England (Figure 2F).

### 3.4 Risk factors for cervical cancer in China, England and India

**Figure 3:**
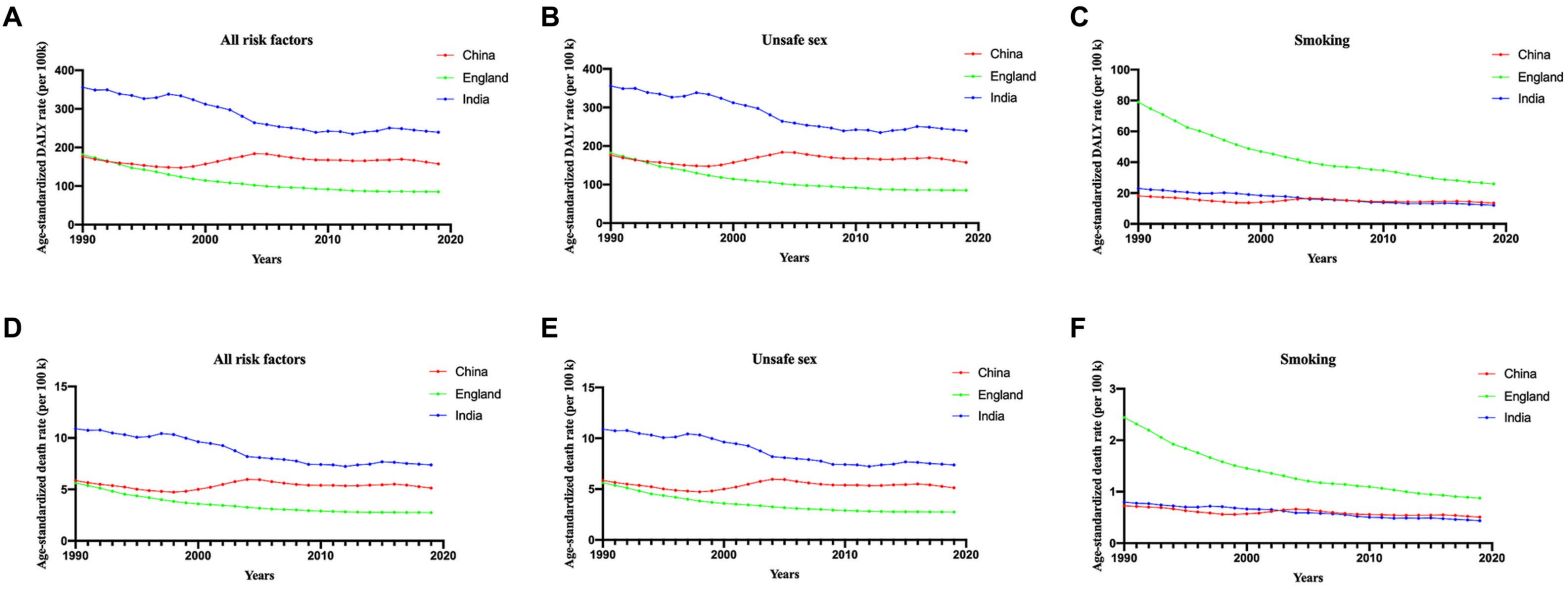
Proportion of DALYs **(A–C)** and ASDR **(D–F)** attributable to all risk factors, unsafe sex and smoking, for China, England and India from 1990 to 2019. ASDR, age-standardized death rate.

To explore potential risk factors, we conducted an analysis encompassing all attributable risk factors within the GBD database (Figure 3). In general, unsafe sex emerged as the most prominent risk factor across the three different countries (Figures 3B,D). The age-standardized DALY rate and ASDR exhibited a declining trend for England and India but fluctuated in the case of China.

Notably, smoking plays a crucial role in both DALY and ASDR in England. However, it is noteworthy that, since 1990, smoking has consistently demonstrated a significant downwards trend, as illustrated in Figures 3C,F. The ASDR and DALY rates attributed to smoking were quite similar between China and India.

### 3.5 Changes in burden, incidence and death in the next ten years

**Figure 4:**
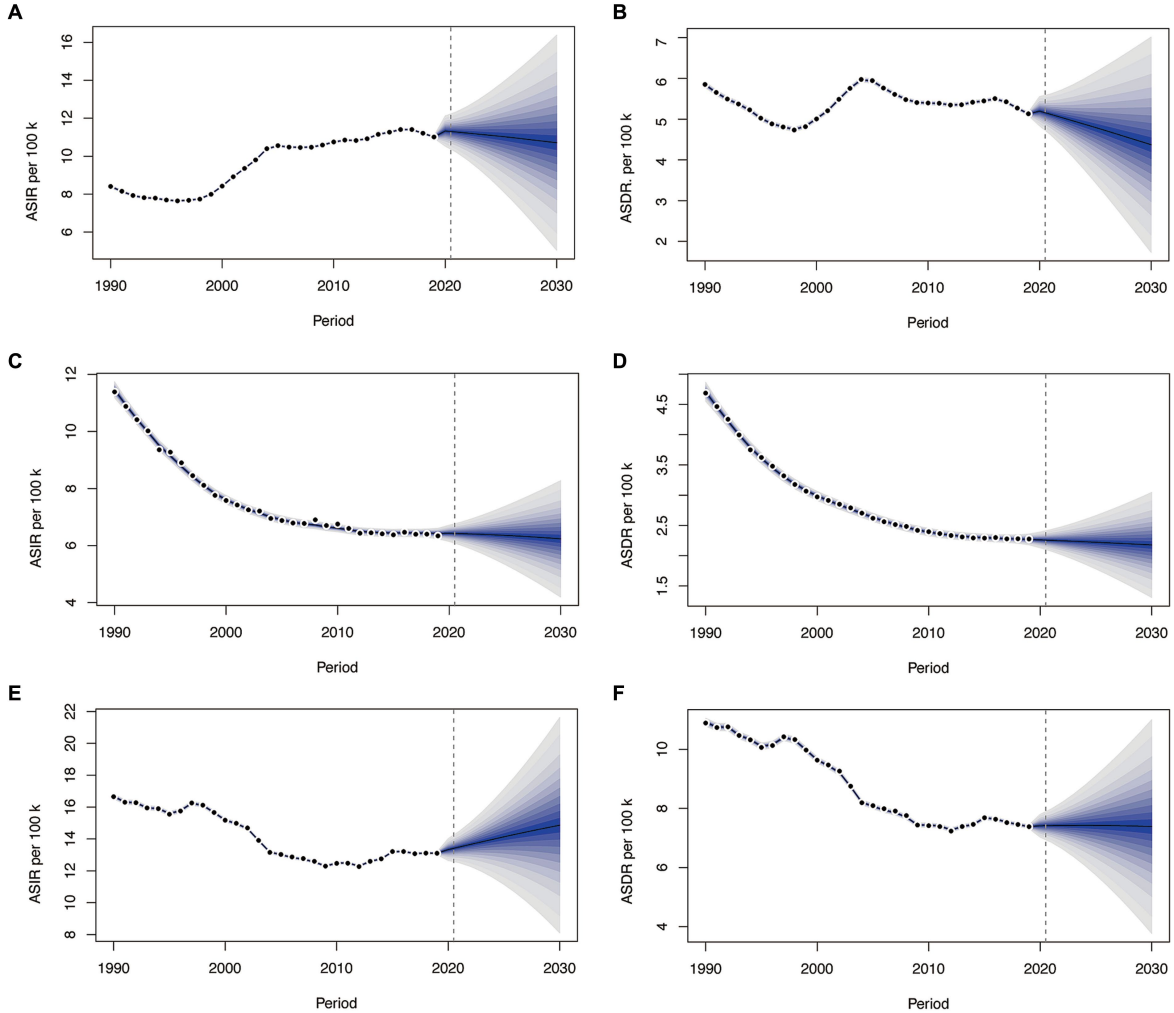
The change trends of the ASIR and ASDR from 1990 to 2030 in China **(A,B)**, England **(C,D)**, and India **(E,F)**. ASIR, age-standardized incidence rate.

From 2019 to 2030, the ASIR in China has exhibited a gradual decline, settling at 10.71/100000, albeit still higher than the 1990 level (Figure 4A). Meanwhile, the ASDR has decreased to 4.37/100000, marking a reduction compared to that in 1990 (Figure 4B). In England, both ASIR and ASDR have consistently displayed a rapid declines over the 30-year span, reaching approximately 6.24/100000 and 2.18/100000 by 2030, respectively (Figures 4C,D). Turning to India, the ASIR exhibited a declining trend from 1990 to 2012, but it began to gradually increases in 2013, reaching 14.86 by 2030 (Figure 4E). The ASDR witnessed a swift decline from 1990 to 2012, followed by a gradual reduction starting in 2015, reaching 7.39/100000 by 2030 (Figure 4F). Morbidity and mortality rates in the England and China will experience a downwards trend over the next decade, while morbidity rates in India are still rising. The GBD Foresight Visualization showed that the ASDR is consistently on a downward trend for the next 20 years (Supplementary Figure 2).

## 4 Discussion

The study comprehensively demonstrated the trend of the burden of cervical cancer by year and age with measurements of incidence, death and DALYs in the past 30 years and compared the differences among China, England, and India from the GBD database. We found that India had the highest rates of incidence, mortality and DALY among the three countries. Furthermore, unsafe sex is the most important risk factor, which deserves our attention. As developing countries, both China and India continue to face with substantial burden of cervical cancer, necessitating the implementation of policies to alleviate it.

Cervical cancer has a relatively high incidence rate in China, possibly due to factors such as a large population, urban–rural disparities (12), and healthcare inequalities. The mortality rate for cervical cancer patients is also relatively high, partly because of uneven distribution of healthcare resources across different regions, resulting in some women not receiving timely treatment. China has implemented a variety of initiatives (13), such as cervical cancer vaccination programs and routine screenings. Nevertheless, regional disparities persist, with urban areas often enjoying more comprehensive coverage while rural and remote areas encounter accessibility challenges.

The incidence of cervical cancer is relatively low in England, which can be attributed to the highly developed healthcare system and widespread screening programs used (14). Similarly, the mortality rate for cervical cancer patients is also relatively low, reflecting effective early diagnosis and treatment. In England, there are well-established cervical cancer screening (15) and vaccination programs (16) that play a pivotal roles in early diagnosis and prevention. These programs are readily accessible across the nation, ensuring that a larger number of women can benefit from them.

As a developing country, India has a higher incidence of cervical cancer, possibly due to healthcare inadequacies, cultural factors, education levels, and poverty. In India, healthcare inequality is common, leading many women to not have access to preventive and treatment services. The mortality rate for cervical cancer patiens is also relatively high. While there is substantial evidence supporting the efficacy of screening as an intervention, large-scale screening initiatives are still relatively rare in India, only 2.6 to 5% of women are being screened by Pap smears (17). Understanding the disease and promoting early screening are the most effective measures for preventing cervical cancer. A lack of awareness about cervical cancer, screening, and prevention methods, coupled with negative attitudes and suboptimal practices, constitute the primary factors contributing to the increased incidence of this disease (18). The Indian government has taken measures to increase cervical cancer vaccination rates (19) and improve screening programs. However, obstacles such as healthcare inequalities and insufficient service coverage continue to pose challenges. Improving healthcare service coverage and raising awareness about cervical cancer are crucial.

Persistent HPV infection is recognized as the main pathogenic factor of cervical cancer (20). More than 99% of cervical cancer cases are linked to specific types of high-risk HPV, including 16, 18, 31, 33, 45, 52, and 58. In addition to their well-known transformative properties, cells infected with HPV play an active role in shaping the local environment, creating a supportive post-infection microenvironment (PIM). This PIM is increasingly acknowledged as a crucial element in viral persistence, propagation, and the progression toward malignancy (21). Since the WHO proposed the global strategic goal of eliminating cervical cancer, significant efforts have been made worldwide. Accelerating the coverage of HPV vaccination contributes to achieving this goal, and the effectiveness of HPV vaccination has been confirmed in various countries (22, 23). HPV vaccines are classified into three types based on the types of HPV they target. The 2-valent HPV vaccines target HPV-16 and-18, the 4-valent HPV vaccines target HPV-16, −18, −6, and-11, and the 9-valent HPV vaccines provide protection against HPV-16, −18, −6, −11, −31, −33, −45, −52, and-58. HPV vaccination is an effective measure for the primary prevention of cervical cancer, targeting the underlying causes of the disease. A study involving 15,637 participants revealed that awareness of HPV and HPV vaccination significantly decreases with decreasing education and varies by race and ethnicity. Specifically, awareness levels tend to be lower among Black and Asian populations than among White populations (24). Currently, since its first approval in 2016 in China, the HPV vaccines immunization coverage is still low, with less than 3% (25) for adolescents and less than 6% for the entire population. Cervical cancer accounts for 10% of cancer deaths among women in India, but the HPV vaccine is not included in India’s universal immunization program (26). And England is different, the HPV bivalent vaccine (Cervarix) immunization was first introduced in England, UK in Sept 1, 2008, subsequently the routine vaccination was offered to girls aged 12–13 years and catch-up programs were offered to those aged 14–18 years, resulting in a significant decrease in cervical cancer incidence among young women (27). Accelerating the roll-out of the HPV vaccine could help improve the disease burden of cervical cancer. In addition, risk factors for cervical cancer include other infection and behavioral factors. Infectious factors include sexually transmitted diseases of chlamydia and genital herpes (28), and human immunodeficiency virus (HIV) infection (29). The behavioral factors included age at first sexual intercourse (30, 31), multiple partners (32), parity (28, 33), smoking status (34, 35), prolonged use of oral contraception, cervix dysplasia. This is consistent with the risk factors we found. Therefore, increasing public health awareness is crucial. We should use various media platforms, including television, radio, and social media, to carry out education and publicity activities on cancer. Tertiary prevention measures for cervical cancer should be fully implemented and strictly implemented. First, cervical cancer development should be prevented by advocating for a healthy lifestyle and by receiving the HPV vaccination. Second, women with sex are encouraged to undergo regular cervical cancer screening, including thin-based cytology (TCT) and high-risk HPV testing. Finally, precancerous lesions should be found and treated as soon as possible in time, and comprehensive treatment methods such as surgical resection, radiotherapy and chemotherapy, and immunization should be adopted for cervical cancer during different periods.

The predictive model indicates that the ASIR and ASDR in China have shown a gradual decline. In India, the ASIR demonstrated a gradual increase, whereas the ASDR experienced a gradual decline. In England, both the ASIR and ASDR consistently exhibited a significant decreases over the past 30 years. However, it’s important to note that for the population forecast data from 2020 to 2030, information is only available only for the United Kingdom, and there is no specific data for England. Consequently, there may be some degree of bias in the ASR predictions derived from the BAPC model. Nevertheless, an overall downwards trend remains evident.

However, the study has several limitations. First, GBD data rely on the healthcare information systems and disease surveillance of individual countries. Different countries and regions may use different standards and methods to collect data, which can lead to data inconsistencies. Second, the BAPC model is influenced by various factors, which could introduce some biases. Interpreting its results requires a multifaceted analysis. Third, GBD data reflect an overall level and does not specifically report the disease burden associated with each pathological type, such as squamous cell carcinoma, adenocarcinoma, and adenosquamous carcinoma. Additionally, it is necessary to translate the research findings into usable data, providing a basis of reference for countries to formulate preventive policies.

In summary, the epidemiological data on cervical cancer in these three countries reflect the influence of different stages of development and healthcare systems. In China, the ASIR of cervical cancer has steadily increased, while the ASDR and age-standardized DALY rate exhibit relatively stable fluctuations. In contrast, England demonstrated a clear and consistent decline in the ASIR, ASDR, and DALY rates of cervical cancer. India, on the other hand, grapples with persistently high rates but shows a declining trend. The primary risk factor remains unsafe sex and smoking, underscoring the need for increased public education and heightened awareness among women. Trends over the next decade suggest that China and India still face a huge burden of cervical cancer. When England has made significant progress, China and India need to take more measures to improve the prevention and control of cervical cancer, especially in poorer and remote regions.

## Data availability statement

The original contributions presented in the study are included in the article/Supplementary material, further inquiries can be directed to the corresponding author.

## Ethics statement

The studies involving humans were approved by this study was approved by the Ethics Committee of Tongji Hospital affiliated to Tongji University, Grant No. K-W-2022-019. The studies were conducted in accordance with the local legislation and institutional requirements. Written informed consent for participation was not required from the participants or the participants’ legal guardians/next of kin in accordance with the national legislation and institutional requirements.

## Author contributions

SW: Software, Validation, Writing – original draft, Conceptualization. JJ: Data curation, Funding acquisition, Writing – review & editing. XY: Formal analysis, Writing – review & editing. YW: Funding acquisition, Project administration, Supervision, Writing – review & editing.

## Footnotes

^1^http://ghdx.healthdata.org/gbd-results-tool

## References

[1] Sung H Ferlay J Siegel RL Laversanne M Soerjomataram I Jemal A. GLOBOCAN estimates of incidence and mortality worldwide for 36 cancers in 185 countries. CA Cancer J Clin. (2020) 71:209–49. doi: 10.3322/caac.21660 33538338

[2] Wright AA Howitt BE Myers AP Dahlberg SE Palescandolo E Van Hummelen P. Oncogenic mutations in cervical cancer: genomic differences between adenocarcinomas and squamous cell carcinomas of the cervix. Cancer. (2013) 119:3776–83. doi: 10.1002/cncr.28288, PMID: 24037752 PMC3972000

[3] Rahangdale L Mungo C O'Connor S Chibwesha CJ Brewer NT. Human papillomavirus vaccination and cervical cancer risk. BMJ. (2022) 379:e070115. doi: 10.1136/bmj-2022-070115 36521855

[4] Das M. WHO launches strategy to accelerate elimination of cervical cancer. Lancet Oncol. (2021) 22:20–1. doi: 10.1016/S1470-2045(20)30729-4 33248466

[5] Perkins RB Wentzensen N Guido RS Schiffman M. Cervical Cancer screening: a review. JAMA. (2023) 330:547–58. doi: 10.1001/jama.2023.13174 37552298

[6] Yao H Yan C Qiumin H Li Z Jiao A Xin L. Epidemiological trends and attributable risk burden of cervical Cancer: an observational study from 1990 to 2019. Int J Clin Pract. (2022) 2022:3356431. doi: 10.1155/2022/3356431 36263235 PMC9546700

[7] Sun K Zheng R Lei L Zhang S Zeng H Wang S. Trends in incidence rates, mortality rates, and age-period-cohort effects of cervical Cancer-China, 2003–2017. China CDC Wkly. (2022) 4:1070–6. doi: 10.46234/ccdcw2022.216, PMID: 36751372 PMC9889234

[8] Guo M Xu J Du J. Trends in cervical cancer mortality in China from 1989 to 2018: an age-period-cohort study and Joinpoint analysis. BMC Public Health. (2021) 21:1329. doi: 10.1186/s12889-021-11401-8, PMID: 34229639 PMC8259057

[9] GBD 2019 Diseases and Injuries Collaborators. Global burden of 369 diseases and injuries in 204 countries and territories, 1990–2019: a systematic analysis for the global burden of disease study 2019. Lancet. (2020) 396:1204–22. doi: 10.1016/S0140-6736(20)30925-9 33069326 PMC7567026

[10] Stevens GA Alkema L Black RE Boerma JT Collins GS Ezzati M. Guidelines for accurate and transparent health estimates reporting: the GATHER statement. Lancet. (2016) 388:e19–23. doi: 10.1016/S0140-6736(16)30388-9, PMID: 27371184

[11] Liu Q He H Yang J Feng X Zhao F Lyu J. Changes in the global burden of depression from 1990 to 2017: findings from the global burden of disease study. J Psychiatr Res. 126:134–40. doi: 10.1016/j.jpsychires.2019.08.002 31439359

[12] Yuan M Zhao X Wang H Hu S Zhao F. Trend in cervical Cancer incidence and mortality rates in China, 2006–2030: a Bayesian age-period-cohort modeling study. Cancer Epidemiol Biomarkers Prev. (2023) 32:825–33. doi: 10.1158/1055-9965.EPI-22-0674 36944168

[13] Wang SM Qiao YL. Implementation of cervical cancer screening and prevention in China--challenges and reality. Jpn J Clin Oncol. (2015) 45:7–11. doi: 10.1093/jjco/hyu188, PMID: 25398583

[14] Mendes D Mesher D Pista A Baguelin M Jit M. Understanding differences in cervical cancer incidence in Western Europe: comparing Portugal and England. Eur J Pub Health. (2018) 28:343–7. doi: 10.1093/eurpub/ckx176, PMID: 29059348

[15] Herbert A. Is cervical screening working? A cytopathologist's view from the United Kingdom. Hum Pathol. (1997) 28:120–6. doi: 10.1016/S0046-8177(97)90094-0, PMID: 9023390

[16] Johnson HC Lafferty EI Eggo RM Louie K Soldan K Waller J. Effect of HPV vaccination and cervical cancer screening in England by ethnicity: a modelling study. Lancet Public Health. (2018) 3:e44–51. doi: 10.1016/S2468-2667(17)30238-4, PMID: 29307388 PMC5765530

[17] Shekhar S Sharma C Thakur S Raina N. Cervical cancer screening: knowledge, attitude and practices among nursing staff in a tertiary level teaching institution of rural India. Asian Pac J Cancer Prev. (2013) 14:3641–5. doi: 10.7314/APJCP.2013.14.6.3641 23886159

[18] Taneja N Chawla B Awasthi AA Shrivastav KD Jaggi VK Janardhanan R. Knowledge, attitude, and practice on cervical Cancer and screening among women in India: a review. Cancer Control. (2021) 28:10732748211010799. doi: 10.1177/10732748211010799 33926235 PMC8204637

[19] Sankaranarayanan R Basu P Kaur P Bhaskar R Singh GB Denzongpa P. Current status of human papillomavirus vaccination in India's cervical cancer prevention efforts. Lancet Oncol. (2019) 20:e637–44. doi: 10.1016/S1470-2045(19)30531-5, PMID: 31674322

[20] Hausen HZ. Papillomaviruses and cancer: from basic studies to clinical application. Nat Rev Cancer. (2002) 2:342–50. doi: 10.1038/nrc798, PMID: 12044010

[21] Yuan Y Cai X Shen F Ma F. HPV post-infection microenvironment and cervical cancer. Cancer Lett. (2021) 497:243–54. doi: 10.1016/j.canlet.2020.10.034 33122098

[22] Goncalves CA Pereira-da-Silva G Silveira R Mayer PCM Zilly A Lopes-Junior LC. Safety, efficacy, and immunogenicity of therapeutic vaccines for patients with high-grade cervical intraepithelial neoplasia (CIN 2/3) associated with human papillomavirus: a systematic review. Cancers (Basel). (2024) 16:672. doi: 10.3390/cancers16030672, PMID: 38339423 PMC10854525

[23] Saeki Y Saito M Irie T Itoh F Enatsu A Komura H. Effectiveness of prophylactic HPV vaccines against cervical abnormalities and HPV infection in Japan: the J-HERS 2021 multicenter study. J Med Virol. (2024) 96:e29413. doi: 10.1002/jmv.29413, PMID: 38314927

[24] Stephens ES Dema E McGee-Avila JK Shiels MS Kreimer AR Shing JZ. Human papillomavirus awareness by educational level and by race and ethnicity. JAMA Netw Open. (2023) 6:e2343325. doi: 10.1001/jamanetworkopen.2023.43325, PMID: 37962885 PMC10646733

[25] Hu S Xu X Zhang Y Liu Y Yang C Wang Y. A nationwide post-marketing survey of knowledge, attitude and practice toward human papillomavirus vaccine in general population: implications for vaccine roll-out in mainland China. Vaccine. (2021) 39:35–44. doi: 10.1016/j.vaccine.2020.11.029, PMID: 33243631

[26] Ray S Mulchandani R Patel P. Demand and willingness to pay for human papilloma virus vaccine for their daughters among mothers in Haryana, India: a contingent valuation study. J Health Serv Res Policy. (2023):13558196231215969. doi: 10.1177/13558196231215969 37994804

[27] Falcaro M Castanon A Ndlela B Checchi M Soldan K Lopez-Bernal J. The effects of the national HPV vaccination programme in England, UK, on cervical cancer and grade 3 cervical intraepithelial neoplasia incidence: a register-based observational study. Lancet. (2021) 398:2084–92. doi: 10.1016/S0140-6736(21)02178-4, PMID: 34741816

[28] Vesco KK Whitlock EP Eder M Burda BU Senger CA Lutz K. Risk factors and other epidemiologic considerations for cervical cancer screening: a narrative review for the U.S. preventive services task force. Ann Intern Med. (2011) 155:698–705, W216. doi: 10.7326/0003-4819-155-10-201111150-00377 22006929

[29] Johnson CA James D Marzan A Armaos M. Cervical Cancer: an overview of pathophysiology and management. Semin Oncol Nurs. (2019) 35:166–74. doi: 10.1016/j.soncn.2019.02.003, PMID: 30878194

[30] Ribeiro AA Costa MC Alves RR Villa LL Saddi VA Carneiro MA. HPV infection and cervical neoplasia: associated risk factors. Infect Agent Cancer. (2015) 10:16. doi: 10.1186/s13027-015-0011-3, PMID: 26244052 PMC4524198

[31] Ruiz AM Ruiz JE Gavilanes AV Eriksson T Lehtinen M Perez G. Proximity of first sexual intercourse to menarche and risk of high-grade cervical disease. J Infect Dis. (2012) 206:1887–96. doi: 10.1093/infdis/jis612, PMID: 23066159

[32] Frumovitz M Sun CC Schover LR Munsell MF Jhingran A Wharton JT. Quality of life and sexual functioning in cervical cancer survivors. J Clin Oncol. (2005) 23:7428–36. doi: 10.1200/JCO.2004.00.3996 16234510

[33] McGraw SL Ferrante JM. Update on prevention and screening of cervical cancer. World J Clin Oncol. (2014) 5:744–52. doi: 10.5306/wjco.v5.i4.744, PMID: 25302174 PMC4129537

[34] Collins S Rollason TP Young LS Woodman CB. Cigarette smoking is an independent risk factor for cervical intraepithelial neoplasia in young women: a longitudinal study. Eur J Cancer. (2010) 46:405–11. doi: 10.1016/j.ejca.2009.09.015, PMID: 19819687 PMC2808403

[35] Plummer M Herrero R Franceschi S Meijer CJ Snijders P Bosch FX. Smoking and cervical cancer: pooled analysis of the IARC multi-centric case--control study. Cancer Causes Control. (2003) 14:805–14. doi: 10.1023/b:caco.0000003811.98261.3e 14682438

