# Supplementary material for: Cervical cancer incidence, mortality, and burden in China: a time-trend analysis and comparison with England and India based on the global burden of disease study 2019
Source: Front Public Health. 2024 Mar 6;12:1358433. doi: 10.3389/fpubh.2024.1358433 (PMC10951371; doi:10.3389/fpubh.2024.1358433)
Supplement: Supplementary file 1 [file Table_1.DOCX]

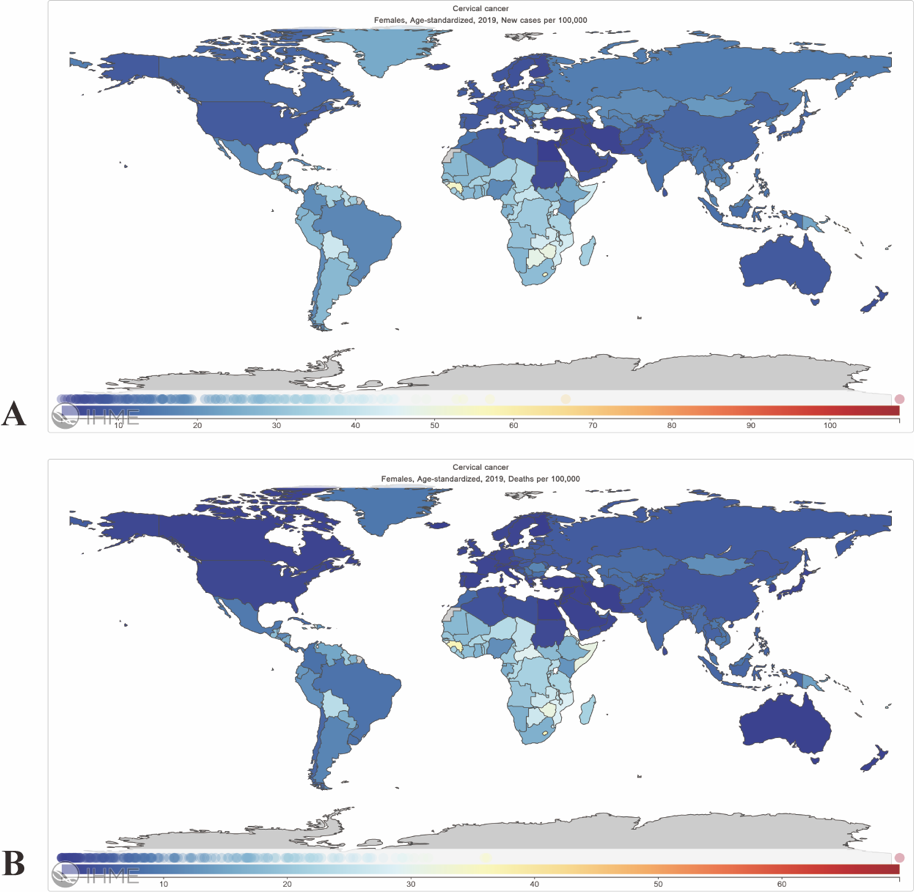


Supplementary Figure 1 Global map of the ASIR (A) and ASDR (B) of cervical cancer from 1990 to 2019. ASIR, age-standardized incidence rate. ASDR, age-standardized death rate.


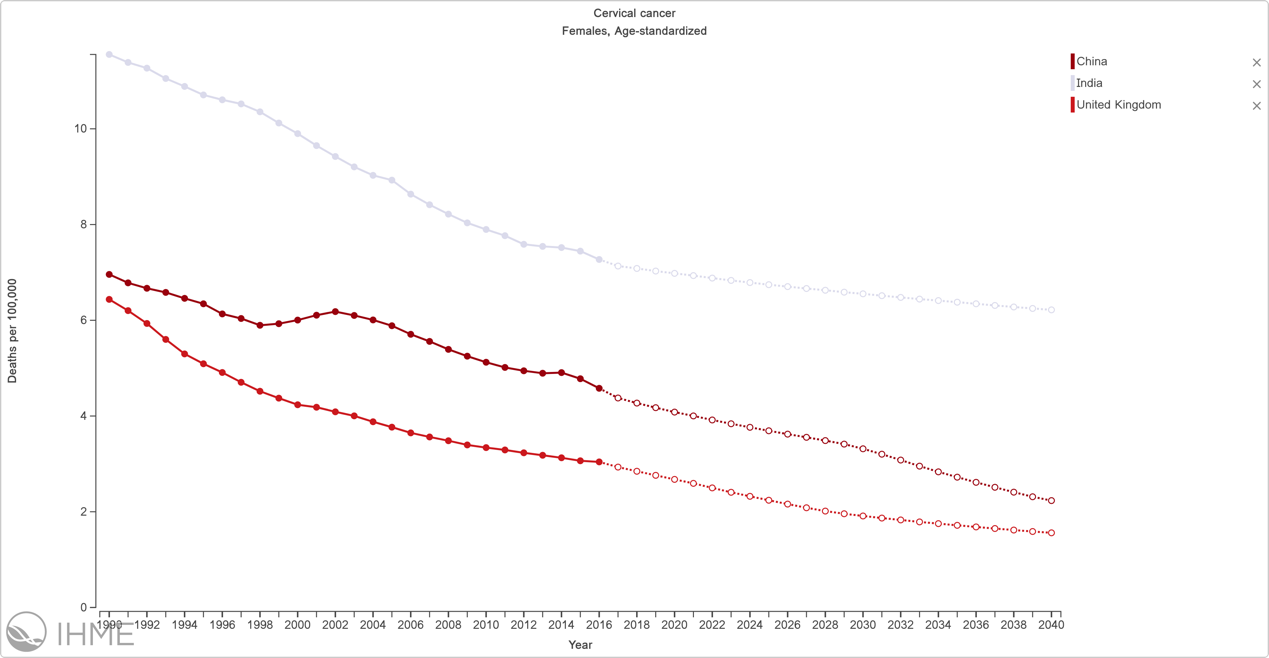


Supplementary Figure 2 The change trends of the ASDR from 1990 to 2040 in China, India and the UK.
